# 4-Bromo-*N*-phenyl­benzamidoxime

**DOI:** 10.1107/S1600536809040057

**Published:** 2009-10-23

**Authors:** Mihaela Cibian, Janaina G. Ferreira, Garry S. Hanan

**Affiliations:** a Département de Chimie, Université de Montréal, CP 6128, Succ. Centre-ville, Montréal, Québec, Canada H3C 3J7

## Abstract

The title compound, C_13_H_11_BrN_2_O, a hydroxy­amidine derivative (an amidoxime), was obtained by addition of the corresponding imidoyl chloride to hydroxy­lamine. The benzene and phenyl rings are twisted from the mean plane of the hydroxy­amidine group by 34.4 (1) and 59.2 (1)°, respectively. In the crystal structure, inter­molecular O—H⋯N hydrogen bonds link pairs of mol­ecules, forming centrosymmetric dimers.

## Related literature

For the synthesis, properties and applications of *N*-substituted hydroxy­amidines/amidoximes see: Krajete *et al.* (2004 ▶), Srivastava *et al.* (1997 ▶); Dondoni *et al.* (1975 ▶, 1977 ▶); Dürüst *et al.* (2000 ▶, 2008 ▶); Exner *et al.* (1974 ▶); Briggs *et al.* (1976 ▶); Deb *et al.* (1991 ▶). For a description of the Cambridge Structural Database, see: Allen *et al.* (1987 ▶).
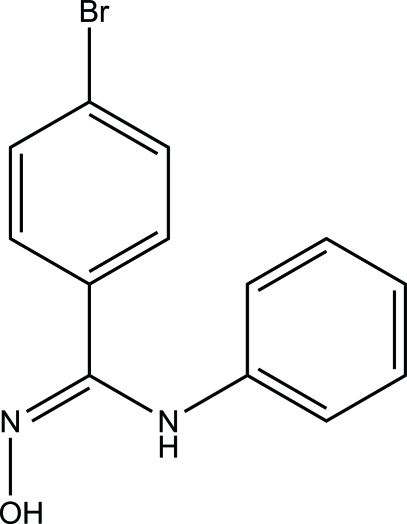


## Experimental

### 

#### Crystal data

C_13_H_11_BrN_2_O
*M* *_r_* = 291.15
Monoclinic, 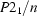

*a* = 6.1752 (1) Å
*b* = 15.2628 (3) Å
*c* = 13.1312 (2) Å
β = 103.415 (1)°
*V* = 1203.86 (4) Å^3^
*Z* = 4
Cu *K*α radiation
μ = 4.53 mm^−1^
*T* = 200 K
0.18 × 0.15 × 0.09 mm

#### Data collection

Bruker APEXII diffractometer
Absorption correction: multi-scan (*SADABS*; Sheldrick, 1996 ▶) *T*_min_ = 0.532, *T*_max_ = 0.665
15615 measured reflections
2356 independent reflections
2273 reflections with *I* > 2σ(*I*)
*R*_int_ = 0.033

#### Refinement

*R*[*F* ^2^ > 2σ(*F* ^2^)] = 0.037
*wR*(*F* ^2^) = 0.090
*S* = 1.11
2356 reflections
155 parameters
H-atom parameters constrained
Δρ_max_ = 0.39 e Å^−3^
Δρ_min_ = −0.68 e Å^−3^

### 

Data collection: *APEX2* (Bruker, 2009 ▶); cell refinement: *SAINT* (Bruker, 2009 ▶); data reduction: *SAINT*; program(s) used to solve structure: *SHELXS97* (Sheldrick, 2008 ▶); program(s) used to refine structure: *SHELXL97* (Sheldrick, 2008 ▶); molecular graphics: *SHELXTL* (Sheldrick, 2008 ▶); software used to prepare material for publication: *UdMX* (Maris, 2004 ▶).

## Supplementary Material

Crystal structure: contains datablocks I, global. DOI: 10.1107/S1600536809040057/lh2914sup1.cif

Structure factors: contains datablocks I. DOI: 10.1107/S1600536809040057/lh2914Isup2.hkl

Additional supplementary materials:  crystallographic information; 3D view; checkCIF report

## Figures and Tables

**Table 1:** Hydrogen-bond geometry (Å, °)

*D*—H⋯*A*	*D*—H	H⋯*A*	*D*⋯*A*	*D*—H⋯*A*
O1—H1⋯N1^i^	0.84	1.99	2.733 (2)	147

Symmetry code: (i) 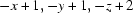.

**Fig. 1.**
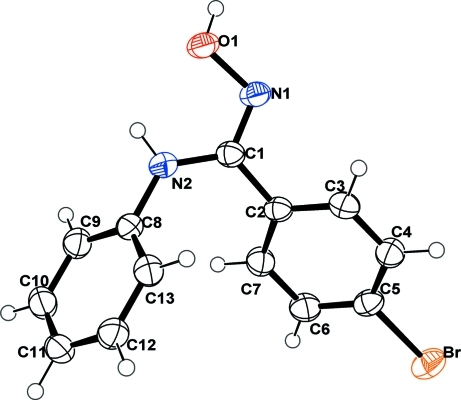
The molecular structure of the title compound (50% probability displacement ellipsoids).

**Fig. 2.**
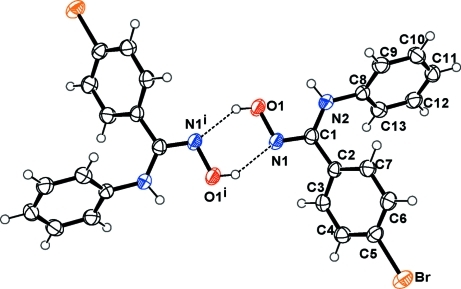
A pair of molecules linked through intermolecular N—H···O bonds [Symmetry code: (i)1 - x,1 - y, 2 - z]. Hydrogen bonds are shown as dashed lines.

## supplementary crystallographic information

### Comment

Although extensively studied for their biological activity (antituberculars, hypotensives), their pharmacological properties (bactericidal, fungicidal, local anaesthetics) (Srivastava, 1997) and also as precursors in the synthesis of cyclic compounds (Dürüst, 2000 and 2008), N-substituted hydroxyamidines/amidoximes have been less investigated concerning their role in coordination and supramolecular chemistry. They act as bidentate ligands to form 5-membered chelate rings with metal ions, forming stable metal complexes. The good electronic delocalization presented by their structures, and the interesting design possibilities, suggest that N-substituted hydroxyamidines/amidoximes and their complexes could be successfully incorporated into supramolecular assemblies based on coordination chemistry and hydrogen bonding. Herein we report the synthesis and crystal structure of a new amidoxime derivative.

The molecular structure of the title compound is shown in Fig. 1. The amidoxime group is present in its neutral form, N—C=N—OH and the bond lengths and angles are within normal ranges (Allen, 1987). The mean planes of the benzene and phenyl rings are tilted with respect to each other by 64.63 (9) ° and, the amidoxime group forms dihedral angles with the benzene and the phenyl rings of 34.4 (1) and 59.2 (1)°, respectively. This value is less than that reported for the bulky substituted *N*-aryl compound (Krajete, 2004) due to the lesser influence of steric crowding in the title compound.

As illustrated in Fig. 2, the hydrogen bond is of crucial importance to the self-assembly. Molecules are paired by two hydrogen bonds involving the *N*-hydroxyl group rather than the amidoxime moieties. In the crystal structure, the *N*-hydroxyl groups participate in hydrogen bonding of the O—H···N type in which two molecules are joined *via* O—H···N hydrogen bonds to form a dimer across an inversion center (Table 1).

### Experimental

The title compound was synthesized according to the procedure of Krajete *et al.* (2004). 4-Bromo-*N*-phenylbenzamide (1.5 g, 5.43 mmol) and an excess of thionyl chloride (15 ml) were refluxed for 2 h under nitrogen atmosphere, yielding the corresponding imidoyl chloirde as a pale yellow solid. This compound was dissolved in dry dichlorometane and added drop-wise to a mixture of hydroxylamine hydrochloride (0.4 g, 5.97 mmol) in anhydrous ethanol and triethylamine (3.8 ml, 27.1 mmol) in dry dichloromethane at 195K. The reaction mixture was brought to room temperature and then was heated at reflux for 16 h. The resulting yellow solution was washed with distillated water and the organic materials were subsequently extracted with diethyl ether, dried over anhydrous Na_2_SO_4_ and filtered. X-ray quality crystals were obtained from a solution of the title compound in aqueous EtOH by slow evaporation at room temperature.

1H NMR (DMSO-d6, 300 MHz, δ, p.p.m.): 10.66 (s, 1H), 8.34 (s, 1H), 7.52(d, *J = *8.4 Hz, 2H), 7.30, (d, *J= *8.4 Hz, 2H), 7.08 (t, *J = *7.8, 7.8 Hz, 2H), 6.80 (t, *J =*7.3, 7.3 Hz, 1H), 6.65 (d, *J = *7.8 Hz, 2H).

### Refinement

The H atoms were generated geometrically (C—H 0.95, N—H 0.88, O—H 0.84 Å) and were included in the refinement in the riding model approximation; their temperature factors were set to 1.5 and 1.2 times for oxygen atom and for those of the equivalent isotropic temperature factors of the parent site, respectively.

### Crystal data

**Table:**

C_13_H_11_BrN_2_O	*F*(000) = 584
*M**_r_* = 291.15	*D*_x_ = 1.606 Mg m^−^^3^
Monoclinic, *P*2_1_/*n*	Cu *K*α radiation, λ = 1.54178 Å
Hall symbol: -P 2yn	Cell parameters from 11676 reflections
*a* = 6.1752 (1) Å	θ = 2.9–71.9°
*b* = 15.2628 (3) Å	µ = 4.53 mm^−^^1^
*c* = 13.1312 (2) Å	*T* = 200 K
β = 103.415 (1)°	Block, yellow
*V* = 1203.86 (4) Å^3^	0.18 × 0.15 × 0.09 mm
*Z* = 4	

### Data collection

**Table:**

Bruker APEXII diffractometer	2356 independent reflections
Radiation source: Rotating Anode	2273 reflections with *I* > 2σ(*I*)
Montel 200 optics	*R*_int_ = 0.033
Detector resolution: 5.5 pixels mm^-1^	θ_max_ = 72.5°, θ_min_ = 4.5°
ω scans	*h* = −7→7
Absorption correction: multi-scan (*SADABS*; Sheldrick, 1996)	*k* = −18→18
*T*_min_ = 0.532, *T*_max_ = 0.665	*l* = −15→16
15615 measured reflections	

### Refinement

**Table:**

Refinement on *F*^2^	Secondary atom site location: difference Fourier map
Least-squares matrix: full	Hydrogen site location: inferred from neighbouring sites
*R*[*F*^2^ > 2σ(*F*^2^)] = 0.037	H-atom parameters constrained
*wR*(*F*^2^) = 0.090	*w* = 1/[σ^2^(*F*_o_^2^) + (0.0601*P*)^2^ + 0.3484*P*] where *P* = (*F*_o_^2^ + 2*F*_c_^2^)/3
*S* = 1.11	(Δ/σ)_max_ = 0.001
2356 reflections	Δρ_max_ = 0.39 e Å^−^^3^
155 parameters	Δρ_min_ = −0.68 e Å^−^^3^
0 restraints	Extinction correction: *SHELXL97* (Sheldrick, 2008), Fc^*^=kFc[1+0.001xFc^2^λ^3^/sin(2θ)]^-1/4^
Primary atom site location: structure-invariant direct methods	Extinction coefficient: 0.0212 (8)

### Special details

**Table:**

Experimental. X-ray crystallographic data for (I) were collected from a single-crystal sample, which was mounted on a loop fiber. Data were collected using a Bruker Platform diffractometer, equipped with a Bruker *SMART* 4 K Charged-Coupled Device (CCD) Area Detector using the program *APEX2* and a Nonius FR591 rotating anode equiped with a Montel 200 optics The crystal-to-detector distance was 5.0 cm, and the data collection was carried out in 512 *x* 512 pixel mode. The initial unit-cell parameters were determined by a least-squares fit of the angular setting of strong reflections, collected by a 10.0 degree scan in 33 frames over four different parts of the reciprocal space (133 frames total). One complete sphere of data was collected, to better than 0.80Å resolution.
Geometry. All e.s.d.'s (except the e.s.d. in the dihedral angle between two l.s. planes) are estimated using the full covariance matrix. The cell e.s.d.'s are taken into account individually in the estimation of e.s.d.'s in distances, angles and torsion angles; correlations between e.s.d.'s in cell parameters are only used when they are defined by crystal symmetry. An approximate (isotropic) treatment of cell e.s.d.'s is used for estimating e.s.d.'s involving l.s. planes.
Refinement. Refinement of *F*^2^ against ALL reflections. The weighted *R*-factor *wR* and goodness of fit *S* are based on *F*^2^, conventional *R*-factors *R* are based on *F*, with *F* set to zero for negative *F*^2^. The threshold expression of *F*^2^ > σ(*F*^2^) is used only for calculating *R*-factors(gt) *etc*. and is not relevant to the choice of reflections for refinement. *R*-factors based on *F*^2^ are statistically about twice as large as those based on *F*, and *R*- factors based on ALL data will be even larger.

### Fractional atomic coordinates and isotropic or equivalent isotropic displacement parameters (Å^2^)

**Table:**

	*x*	*y*	*z*	*U*_iso_*/*U*_eq_	
Br	1.22913 (4)	0.643427 (14)	0.618702 (18)	0.04819 (16)	
O1	0.4208 (2)	0.40817 (9)	0.94989 (11)	0.0393 (3)	
H1	0.3752	0.4375	0.9949	0.059*	
N1	0.5789 (3)	0.45874 (10)	0.91086 (12)	0.0340 (3)	
N2	0.4624 (3)	0.36287 (11)	0.77216 (14)	0.0410 (4)	
H2	0.3431	0.3517	0.7958	0.049*	
C1	0.5957 (3)	0.42884 (11)	0.82101 (14)	0.0313 (4)	
C2	0.7549 (3)	0.47517 (11)	0.77017 (13)	0.0299 (4)	
C3	0.9510 (3)	0.50983 (12)	0.83173 (14)	0.0346 (4)	
H3	0.9873	0.4994	0.9051	0.041*	
C4	1.0932 (3)	0.55912 (13)	0.78747 (15)	0.0368 (4)	
H4	1.2261	0.5828	0.8299	0.044*	
C5	1.0391 (3)	0.57343 (12)	0.68075 (15)	0.0353 (4)	
C6	0.8484 (4)	0.53857 (14)	0.61731 (15)	0.0408 (4)	
H6	0.8153	0.5479	0.5438	0.049*	
C7	0.7060 (3)	0.48970 (13)	0.66248 (15)	0.0381 (4)	
H7	0.5738	0.4659	0.6196	0.046*	
C8	0.4934 (3)	0.31015 (12)	0.68770 (14)	0.0337 (4)	
C9	0.3113 (3)	0.29434 (13)	0.60544 (16)	0.0386 (4)	
H9	0.1703	0.3189	0.6062	0.046*	
C10	0.3362 (4)	0.24228 (13)	0.52181 (16)	0.0417 (4)	
H10	0.2117	0.2309	0.4656	0.050*	
C11	0.5407 (4)	0.20724 (13)	0.52029 (16)	0.0414 (4)	
H11	0.5577	0.1726	0.4626	0.050*	
C12	0.7220 (3)	0.22247 (14)	0.60292 (17)	0.0418 (5)	
H12	0.8629	0.1980	0.6017	0.050*	
C13	0.6989 (3)	0.27319 (13)	0.68740 (15)	0.0384 (4)	
H13	0.8226	0.2826	0.7446	0.046*	

### Atomic displacement parameters (Å^2^)

**Table:**

	*U*^11^	*U*^22^	*U*^33^	*U*^12^	*U*^13^	*U*^23^
Br	0.0483 (2)	0.0459 (2)	0.0568 (2)	−0.00414 (8)	0.02515 (13)	0.00910 (8)
O1	0.0486 (8)	0.0365 (7)	0.0400 (7)	−0.0037 (6)	0.0248 (6)	−0.0008 (5)
N1	0.0401 (8)	0.0326 (8)	0.0336 (7)	0.0003 (6)	0.0173 (6)	0.0020 (6)
N2	0.0419 (9)	0.0438 (9)	0.0439 (10)	−0.0102 (7)	0.0235 (8)	−0.0114 (7)
C1	0.0353 (8)	0.0289 (8)	0.0315 (8)	0.0025 (7)	0.0113 (7)	−0.0001 (6)
C2	0.0346 (8)	0.0272 (8)	0.0300 (8)	0.0029 (6)	0.0118 (7)	0.0004 (6)
C3	0.0399 (9)	0.0362 (9)	0.0280 (8)	0.0020 (7)	0.0088 (7)	0.0005 (7)
C4	0.0353 (9)	0.0370 (9)	0.0380 (9)	−0.0008 (7)	0.0086 (7)	−0.0007 (7)
C5	0.0383 (9)	0.0304 (8)	0.0409 (10)	0.0017 (7)	0.0170 (7)	0.0038 (7)
C6	0.0492 (11)	0.0437 (10)	0.0298 (9)	−0.0029 (9)	0.0101 (8)	0.0063 (8)
C7	0.0422 (10)	0.0403 (10)	0.0306 (9)	−0.0052 (8)	0.0061 (7)	0.0018 (7)
C8	0.0394 (9)	0.0307 (8)	0.0344 (9)	−0.0060 (7)	0.0156 (7)	−0.0028 (7)
C9	0.0377 (9)	0.0352 (9)	0.0439 (10)	−0.0029 (7)	0.0113 (8)	−0.0017 (8)
C10	0.0479 (11)	0.0383 (10)	0.0373 (10)	−0.0075 (8)	0.0065 (8)	−0.0035 (8)
C11	0.0565 (12)	0.0332 (9)	0.0389 (10)	−0.0079 (8)	0.0199 (9)	−0.0072 (7)
C12	0.0410 (10)	0.0356 (10)	0.0534 (11)	−0.0016 (7)	0.0203 (9)	−0.0058 (8)
C13	0.0374 (9)	0.0372 (9)	0.0409 (10)	−0.0031 (7)	0.0099 (8)	−0.0046 (8)

### Geometric parameters (Å, °)

**Table:**

Br—C5	1.9040 (18)	C6—C7	1.387 (3)
O1—N1	1.430 (2)	C6—H6	0.9500
O1—H1	0.8400	C7—H7	0.9500
N1—C1	1.292 (2)	C8—C9	1.388 (3)
N2—C1	1.363 (2)	C8—C13	1.390 (3)
N2—C8	1.419 (2)	C9—C10	1.393 (3)
N2—H2	0.8800	C9—H9	0.9500
C1—C2	1.489 (2)	C10—C11	1.376 (3)
C2—C7	1.394 (2)	C10—H10	0.9500
C2—C3	1.394 (3)	C11—C12	1.386 (3)
C3—C4	1.383 (3)	C11—H11	0.9500
C3—H3	0.9500	C12—C13	1.387 (3)
C4—C5	1.381 (3)	C12—H12	0.9500
C4—H4	0.9500	C13—H13	0.9500
C5—C6	1.382 (3)		
			
N1—O1—H1	109.5	C7—C6—H6	120.5
C1—N1—O1	110.06 (15)	C6—C7—C2	120.66 (17)
C1—N2—C8	127.65 (17)	C6—C7—H7	119.7
C1—N2—H2	116.2	C2—C7—H7	119.7
C8—N2—H2	116.2	C9—C8—C13	120.19 (17)
N1—C1—N2	121.56 (17)	C9—C8—N2	118.38 (18)
N1—C1—C2	116.31 (16)	C13—C8—N2	121.42 (17)
N2—C1—C2	121.95 (16)	C8—C9—C10	119.66 (19)
C7—C2—C3	118.85 (16)	C8—C9—H9	120.2
C7—C2—C1	121.35 (16)	C10—C9—H9	120.2
C3—C2—C1	119.67 (15)	C11—C10—C9	120.21 (19)
C4—C3—C2	120.89 (17)	C11—C10—H10	119.9
C4—C3—H3	119.6	C9—C10—H10	119.9
C2—C3—H3	119.6	C10—C11—C12	120.06 (18)
C5—C4—C3	119.02 (17)	C10—C11—H11	120.0
C5—C4—H4	120.5	C12—C11—H11	120.0
C3—C4—H4	120.5	C11—C12—C13	120.36 (19)
C4—C5—C6	121.51 (17)	C11—C12—H12	119.8
C4—C5—Br	119.69 (15)	C13—C12—H12	119.8
C6—C5—Br	118.80 (14)	C12—C13—C8	119.50 (18)
C5—C6—C7	119.05 (17)	C12—C13—H13	120.3
C5—C6—H6	120.5	C8—C13—H13	120.3
			
O1—N1—C1—N2	4.8 (2)	Br—C5—C6—C7	−178.10 (16)
O1—N1—C1—C2	179.94 (14)	C5—C6—C7—C2	−0.5 (3)
C8—N2—C1—N1	−164.13 (19)	C3—C2—C7—C6	−0.7 (3)
C8—N2—C1—C2	21.0 (3)	C1—C2—C7—C6	175.18 (18)
N1—C1—C2—C7	−141.09 (18)	C1—N2—C8—C9	−135.4 (2)
N2—C1—C2—C7	34.0 (3)	C1—N2—C8—C13	46.0 (3)
N1—C1—C2—C3	34.7 (2)	C13—C8—C9—C10	−0.9 (3)
N2—C1—C2—C3	−150.13 (18)	N2—C8—C9—C10	−179.53 (17)
C7—C2—C3—C4	1.1 (3)	C8—C9—C10—C11	−0.5 (3)
C1—C2—C3—C4	−174.82 (16)	C9—C10—C11—C12	1.1 (3)
C2—C3—C4—C5	−0.3 (3)	C10—C11—C12—C13	−0.2 (3)
C3—C4—C5—C6	−1.0 (3)	C11—C12—C13—C8	−1.3 (3)
C3—C4—C5—Br	178.52 (14)	C9—C8—C13—C12	1.8 (3)
C4—C5—C6—C7	1.4 (3)	N2—C8—C13—C12	−179.64 (18)

### Hydrogen-bond geometry (Å, °)

**Table:**

*D*—H···*A*	*D*—H	H···*A*	*D*···*A*	*D*—H···*A*
O1—H1···N1^i^	0.84	1.99	2.733 (2)	147

Symmetry codes: (i) −*x*+1, −*y*+1, −*z*+2.
